# FewMedical-XJAU: A Challenging Benchmark for Fine-Grained Medicinal Plant Classification

**DOI:** 10.3390/s25175499

**Published:** 2025-09-04

**Authors:** Tao Zhang, Sheng Huang, Gulimila Kezierbieke, Yeerjiang Halimu, Hui Li

**Affiliations:** 1 College of Computer and Information Engineering, Xinjiang Agricultural University, Urumqi 830052, China; 320233431@xjau.edu.cn (T.Z.); yej@xjau.edu.cn (Y.H.); 2 School of Artificial Intelligence and Computer Science, Jiangnan University, Wuxi 214122, China; 6243111031@stu.jiangnan.edu.cn (S.H.); lihui.cv@jiangnan.edu.cn (H.L.)

**Keywords:** fine-grained classification, dynamic fusion, few-shot, dataset

## Abstract

Fine-grained plant image classification (FPIC) aims to distinguish plant species with subtle visual differences, but existing datasets often suffer from limited category diversity, homogeneous backgrounds, and insufficient environmental variation, limiting their effectiveness in complex real-world scenarios. To address these challenges, a novel dataset, FewMedical-XJAU, is presented, focusing on rare medicinal plants native to Xinjiang, China. This dataset offers higher intra-class variability, more complex and diverse natural backgrounds, varied shooting angles and lighting conditions, and more rigorous expert annotations, providing a realistic testbed for FPIC tasks. Building on this, an improved method called BDCC (Bilinear Deep Cross-modal Composition) is proposed, which incorporates textual priors into a deep metric learning framework to enhance semantic discrimination. A Class-Aware Structured Text Prompt Construction strategy is introduced to improve the model’s semantic understanding, along with a dynamic fusion mechanism to address high inter-class similarity and intra-class variability. In few-shot classification experiments, the method demonstrates superior accuracy and robustness under complex environmental conditions, offering strong support for practical applications of fine-grained classification.

## 1. Introduction

Medicinal plants play an irreplaceable role in traditional medicine due to their unique therapeutic properties. According to recent statistics, among the 2579 known medicinal plant species worldwide, 13% are listed as threatened on the International Union for Conservation of Nature (IUCN) Red List (2020) [1]. This global trend underscores the urgency of conserving wild medicinal plants in regions such as Xinjiang. Notably, since the 1990s, over 60% of China’s grasslands have experienced degradation, compared with only 10% prior to the 1970s, and the rate of degradation continues to accelerate. In Northwest China, up to 95% of natural grasslands have undergone desertification or salinization as a result of environmental changes and anthropogenic activities [2]. Such ecological degradation not only severely threatens the survival and development of local endangered medicinal plants but also poses significant challenges to socio-economic systems that rely on natural ecosystems.

Furthermore, the region harbors several rare medicinal plant species. Without timely and targeted conservation measures, many of these species face the risk of permanent extinction [3]. Therefore, accurate identification and classification of medicinal plants are essential for both ecological conservation and the advancement of medical research.

Deep learning models provide strong support for efficient identification and classification of plant species. However, the accuracy of these models largely depends on the characteristics of the datasets [4,5].

Early benchmark datasets such as Flavia (Wu et al., 2007) [6] included only leaf images of 32 plant species with plain backgrounds. The ImageCLEF 2013 (Caputo et al. 2013) [7] dataset expanded the number of categories to 250 and introduced diversity in backgrounds and plant organs, yet the backgrounds remained relatively simple and the classification hierarchy limited. Pl@ ntNet-300K (Garcin et al., 2021) [8] significantly improved data diversity, but the image quality varied, annotation information was limited, and background complexity was still insufficient.

Although plant image databases have demonstrated multidimensional technical advancements in their design and construction, challenges remain. In terms of data acquisition, early datasets such as Flavia (Wu et al., 2007) [6], Swedish Leaf ((Söderkvist et al. 2001) [9], and LeafSnap (Kumar et al., 2012 ) [10] relied on uniform white backgrounds and single-organ (leaf) imaging, ensuring feature visibility and annotation consistency. ImageCLEF 2013 (Caputo et al., 2013) [7] introduced a dual classification scheme by adding plant organ types alongside species labels, and differentiated between plain and simple scene backgrounds, thereby enhancing data diversity. Further, Pl@ ntNet-300K (Garcin et al., 2021) [8] aggregated 300,000 images from worldwide distributions, covering over a thousand species, and significantly increased background complexity, although it still lacked wide-angle field-of-view samples. Among medicinal plant datasets, MED117 (Sarma et al., 2023) [11] expanded intra-class samples by extracting frames from videos, but backgrounds and viewpoints remained highly consistent; the Bangladeshi medicinal plant dataset (Borkatulla et al., 2023) [12] incorporated multiple leaf images to enrich morphological information; DIMPSAR (Pushpa et al., 2023) [13] added whole-plant images; and Karst Landform Herbs (Tang et al., 2025) [14] uniquely introduced complex natural backgrounds within medicinal datasets, combined with a dual-label system encompassing species and plant organs.

Despite these advancements, database construction faces several challenges. Some datasets exhibit insufficient species coverage, intra-class variability, and ecological diversity, limiting model generalization capabilities. Background complexity is polarized—either overly simplistic, failing to simulate real-world environments, or excessively cluttered in natural scenes, introducing noise. Annotation schemes vary significantly, ranging from flat species-level labels to multi-layered metadata annotations, but lack standardization; moreover, crowdsourced datasets are prone to mislabeling. Even expert-annotated datasets suffer from inadequate inter-expert consistency and deficient long-term version control, impeding cross-database integration and reproducible research.

An ideal plant image database should achieve comprehensive optimization in terms of species coverage, environmental diversity, temporal span, annotation schema, and quality control to meet the high standards required for fine-grained recognition and classification tasks.

1. Species Coverage and Ecological Representativeness
  The database should encompass a wide range of plant species, including common, rare, endangered, and endemic taxa, supporting biodiversity monitoring, ecological conservation, and interdisciplinary research.
2. Environmental and Viewpoint Diversity
  Images should be collected under diverse natural backgrounds, capturing variations in lighting, climate, and terrain to reflect authentic plant habitats and improve model generalization in complex environments. Multiple viewpoints (e.g., top, side, close-up, wide-angle) and scales (organ-level, individual-level, community-level) should be provided.
3. Temporal Dimension and Seasonal Variation
  Data acquisition should span multiple seasons and time points to document the complete plant life cycle from germination to senescence, capturing dynamic changes in morphology, color, and structure, thereby facilitating phenotypic analysis and time-series modeling.
4. Scientific Annotation and Multimodal Extension
  Annotations must adhere to strict botanical taxonomic standards, including hierarchical labels at least down to species and family levels, with fine-grained tags for plant parts and developmental stages when applicable. Multimodal metadata such as textual descriptions, structured attributes (e.g., leaf shape, flower color), geographic coordinates, and imaging conditions should be included to enrich contextual features for machine learning models.
5. Data Quality Control and Sustainable Management
  Data collection should ensure image clarity and consistent exposure; annotations require multiple rounds of expert review and cross-validation to minimize errors and ambiguities. The database should implement version control and update mechanisms to ensure traceability and long-term usability, while providing open interfaces to facilitate academic and industrial sharing and reproducibility.

**Figure 1:**
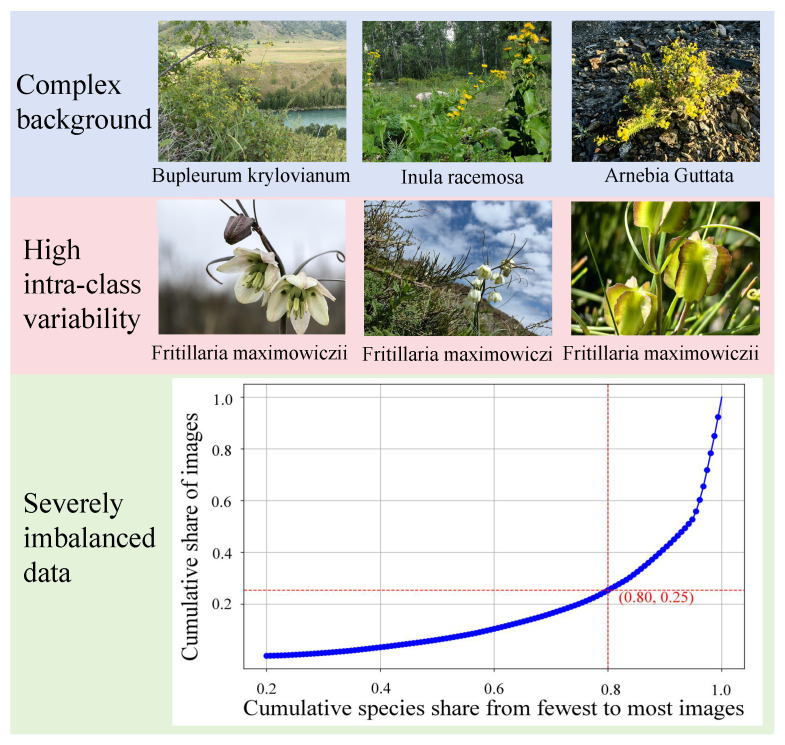
The figure illustrates representative samples and the inherent characteristics of rare data in the dataset. The first row shows representative images with complex backgrounds, while the second row presents images exhibiting high intra-class variability. In the third row, the horizontal axis represents the proportion of plant species, and the vertical axis represents the proportion of plant images. The red point at coordinates (0.8, 0.25) indicates that 80% of the species account for only 25% of the total images. This reflects a typical long-tail distribution, which corresponds to the inherent scarcity of rare plants and naturally aligns with the research paradigm of few-shot learning.

To address the aforementioned challenges and provide a benchmark, we constructed a rare medicinal plant image dataset named FewMedical-XJAU. The dataset features complex and varied backgrounds, rich species diversity, standardized annotations, and distinctive characteristics of the Xinjiang region. It contains a total of 4992 images covering 540 plant species, captured under diverse environments and from multiple angles. Figure 1 shows representative samples and the inherent characteristics of rare data in the dataset.

With the rise of Convolutional Neural Networks (CNNs), feature extraction for plant images has gradually shifted from handcrafted methods to automated deep learning approaches. Early studies mainly combined global and local features while optimizing model architectures to balance performance and computational cost. In recent years, various deep learning methods have been widely applied to plant classification, including CNN-based hybrid feature models, lightweight network designs, CNNs enhanced with optimization algorithms, and hybrid approaches combining handcrafted and deep features. Meanwhile, Transformer architectures and their variants, such as Vision Transformer (ViT), have demonstrated strong potential in large-scale and complex tasks.

Moreover, for rare or endangered plants with extremely limited samples, few-shot and zero-shot learning methods have become a research focus. Existing approaches primarily include transfer learning strategies and meta-learning frameworks. Transfer learning performs well when domain discrepancies are small, but its knowledge transfer efficiency drops significantly when the source and target domains differ greatly; meta-learning methods excel in rapid task adaptation but still exhibit limitations in capturing local fine-grained details in fine-grained classification scenarios.

At the same time, multimodal approaches have introduced new paradigms for cross-modal knowledge transfer. A representative example is the CLIP [15] model, which, through large-scale image–text contrastive learning, maps visual and textual data into a shared semantic space, demonstrating strong generalization capabilities in zero-shot and fine-grained classification tasks. Nevertheless, existing CLIP-based methods for plant recognition remain constrained by insufficient semantic richness of category labels, a lack of contextual information, and inadequate characterization of subtle morphological differences under complex backgrounds. Particularly when dealing with plant categories exhibiting “large intra-class variation and small inter-class differences,” there remains significant room for improvement in both accuracy and stability.

To address the challenges in plant recognition—namely, large intra-class variation, small inter-class differences, complex natural scene backgrounds, and scarce samples—this paper proposes a few-shot classification framework based on bimodal collaborative enhancement, termed BDCC. This method integrates the covariance modeling advantages of DeepBDC with the semantic priors of CLIP [15] through multimodal feature alignment and task-adaptive fusion, thereby enhancing discriminative power and robustness in fine-grained classification. BDCC employs structured textual prompts to generate category descriptions from multiple perspectives, including appearance and growth habits, aligning these with visual features in a shared space. This effectively mitigates confusion caused by complex backgrounds and visually similar categories, and dynamically allocates weights to visual and textual modalities based on their performance within each task, ensuring stable accuracy across diverse scenarios. Experimental results demonstrate that the proposed design effectively improves accuracy in fine-grained few-shot plant image classification, achieving the intended objectives.

The main contributions of this study are as follows:

(1) A rare medicinal plant image dataset from Xinjiang is constructed, highlighting its unique ecological and regional characteristics. Owing to species scarcity, the dataset is naturally suited for few-shot learning research.
(2) The images include complex backgrounds, multi-view angles, and seasonal variations, with large intra-class diversity and small inter-class differences, providing a solid foundation for fine-grained recognition in real-world conditions.
(3) A novel few-short learning framework that integrates image and text features is proposed, built on transfer learning. A class-aware structured text prompt method and an adaptive fusion strategy are introduced to enhance recognition robustness and accuracy.

## 2. Related Work

In this section, we review the current mainstream datasets for non-medicinal and medicinal plants, and elaborate on their respective limitations. Table 1 provides a detailed comparison between FewMedical-XJAU and existing datasets. Additionally, two major categories of approaches in few-shot learning are reviewed: transfer learning-based strategies and metric learning-based algorithms, with metric learning further discussed within the broader meta-learning framework, as detailed in Section 2.3.2.

### 2.1. Non-Medicinal Plant Datasets

**Figure 2:**
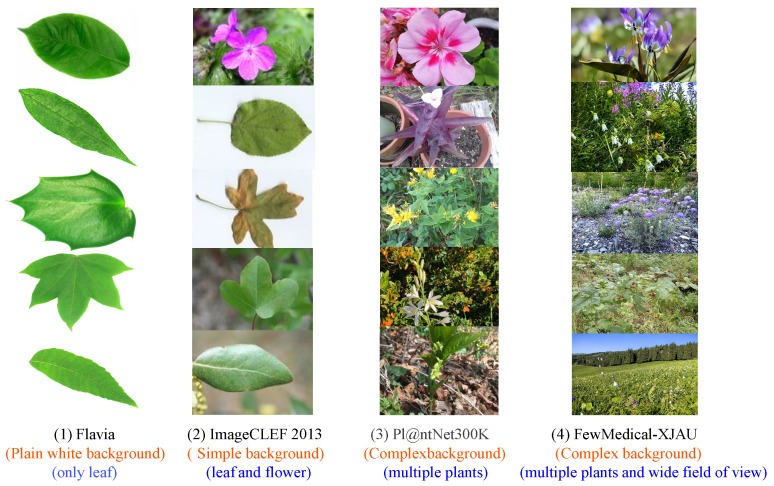
This figure presents a comparison of typical images from other plant datasets and the FewMedical-XJAU dataset. The red text highlights the background characteristics of each dataset, including a plain white background, simple backgrounds, and complex backgrounds. The visual content of the plant images is marked in blue font, specifying features such as leaf-only images, images containing both flowers and leaves, multiple plants, and wide field of view.

Flavia (Wu et al., 2007) [6] collects 1907 leaf images from 32 plant species, serving as a benchmark dataset for plant leaf classification and recognition. However, it has several limitations: the number of species is relatively small, it contains only leaf images, and the images are taken under highly controlled conditions with a plain white background [16], as shown in the first column of Figure 2 and the first row of Table 1. Swedish Leaf (Söderkvist et al., 2001) [9] and LeafSnap (Kumar et al., 2012) [10] share similar characteristics with Flavia (Wu et al., 2007) [6].

Building on this foundation, ImageCLEF 2013 (Caputo et al., 2013) [7] achieves a significant advancement in plant classification tasks. As shown in the second row of Table 1, it comprises 26,077 observations from 250 plant species native to France, representing substantial improvements in both scale and representativeness. A key innovation of ImageCLEF 2013 (Caputo et al., 2013) [7] lies in its introduction of a dual classification dimension—background type and plant organ. Specifically, background types include plain white backgrounds (42%) and simple natural backgrounds (58%), while plant organs cover leaves, flowers, fruits, and other morphological features, thereby improving the diversity and applicability of the data. This is illustrated in the second column of Figure 2. However, ImageCLEF 2013 (Caputo et al., 2013) [7] still has certain limitations: the image backgrounds remain simple, most images are close-ups of plants, and many are specimen photos without complex backgrounds.

Despite these improvements, ImageCLEF 2013 (Caputo et al., 2013) [7] still has notable limitations. The image backgrounds remain relatively simple, with most photographs being close-up shots of plants or preserved specimens, lacking complex environmental contexts. Additionally, the classification hierarchy is coarse, offering only species-level labels, which restricts its utility in fine-grained plant classification tasks [17].

Pl@ ntNet-300K (Garcin et al., 2021) [8] aggregates 300,000 plant images from around the world, covering 1081 species, representing a significant improvement over the ImageCLEF 2013 (Caputo et al., 2013) [7] dataset in terms of both species diversity and image quantity. The complexity of image backgrounds also increases notably, moving beyond simple backgrounds (as shown in the third column of Figure 2 and the third row of Table 1). However, Pl@ ntNet-300K (Garcin et al., 2021) [8] contains virtually no images with particularly wide fields of view, whereas the FewMedical-XJAU dataset includes some samples with larger fields of view (see the fourth column of Figure 2). In the domain of image classification, larger fields of view increase background interference and target localization difficulty, posing higher demands on models’ feature extraction and discrimination capabilities. Nevertheless, Pl@ ntNet-300K (Garcin et al., 2021) [8] maintains an irreplaceable advantage in species coverage and dataset scale.

### 2.2. Medicinal Herb Plant Dataset

**Figure 3:**
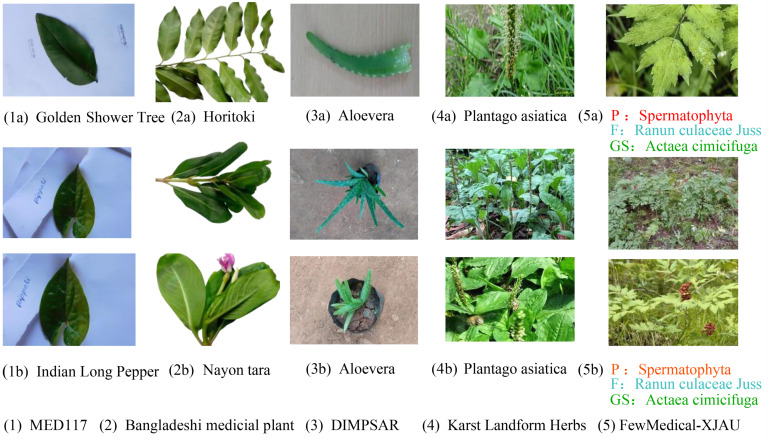
Comparison of typical images between other medicinal plant datasets and FewMedical-XJAU. In the figure, “P” denotes the phylum level, “F” represents the family level, and “GS” refers to the genus and species levels. To illustrate the characteristics of each dataset, a random sampling strategy was employed. For the MED117 and Bangladeshi Medical Plan datasets, where images are presented on a plain white background, samples were randomly selected from all categories within each dataset. For the DIMPSAR, Karst Landform Herbs, and FewMedical-XJAU datasets, which feature complex backgrounds and high intra-class variability, one category was randomly chosen from each dataset, and three images were subsequently randomly selected from the chosen category. The entire sampling process was implemented programmatically to ensure both randomness and representativeness of the samples.

MED117 (Sarma et al., 2023) [11] records 10–15 s videos of each species’ leaves using an SLR Canon camera, with all videos captured against a white background. Still images are then extracted frame-by-frame from the videos, yielding approximately 77,700 images. As shown in Figure 3(1b), since the images derive from video frames, most images from the same plant are morphologically very similar, with uniform backgrounds and each image containing only a single leaf, lacking diversity as illustrated in Figure 3(1a).

The Bangladeshi medicinal plant dataset ((Borkatulla et al., 2023) [12] contains morphologically diverse plant images captured under good lighting conditions, as shown in Figure 3(2b). Compared to MED117 (Sarma et al., 2023) [11], where each image includes only one leaf, this dataset features images with multiple leaves from the same plant (Figure 3(2a)), providing richer morphological information. However, it has limited species coverage and still uses plain white backgrounds.

DIMPSAR (Pushpa et al., 2023) [13] further improves upon the previous two datasets by including 40 medicinal plant species and 5900 images, encompassing both leaf images and full-plant images. Despite these advances, the backgrounds remain simple, and the dataset exhibits low intra-class variation but high inter-class variation, as shown in Figure 3(3b), which may negatively affect model generalization.

The Karst Landform Herbs (Tang et al., 2025) [14] is another representative dataset (see the fourth column of Figure 3). Its main feature is that all images have complex natural backgrounds, and it captures plants in different morphological forms to ensure high intra-class variability. Although this dataset excels in image quality and diversity, its species coverage is limited, as shown in Table 1.

Overall, these datasets exhibit significant differences in image content, background complexity, and intra-class variability, as well as distinct characteristics in annotation methods and quality control. We categorize the annotation approaches of the nine datasets into three groups based on their sources and structure.

Flavia (Wu et al., 2007) [6], Swedish Leaf (Söderkvist et al., 2001) [9], MED117 (Sarma et al., 2023) [11], and the Bangladeshi medicinal plant dataset (Borkatulla et al., 2023 ) [12] are manually annotated by researchers or collection teams with botanical expertise. Their annotation standards are species-based, using a flat, single-layer structure where each class corresponds to a species. These datasets typically rely on uniform collection conditions (e.g., white or plain backgrounds, scanning fresh leaves) to reduce background interference and enhance feature visibility. Quality assurance mainly focuses on standardized collection and imaging protocols, although public documentation generally does not describe cross-validation by independent experts.

LeafSnap (Kumar et al., 2012) [10], ImageCLEF 2013 (Caputo et al., 2013) [7], and Pl@ ntNet-300K (Garcin et al., 2021) [8] employ a combination of crowdsourced data and community or expert verification. Initial data are uploaded by public users, then filtered through collaborative platform mechanisms such as voting, rating, community confirmation, or expert review. Annotation standards are species-based and may include additional metadata such as plant organ types and shooting angles in some datasets. Quality control relies on multi-level validation processes.

DIMPSAR (Pushpa et al., 2023) [13] and Karst Landform Herbs (Tang et al., 2025) [14] are annotated by certified botanical experts, with more rigorous standards. Karst Landform Herbs (Tang et al., 2025) [14] adopts a dual-label system combining species and plant organ (e.g., “species_organ”). To ensure consistency, these datasets involve independent annotations by multiple experts and collective discussions to resolve disagreements. Quality assurance depends on expert identification and cross-validation, ensuring high scientific reliability and accuracy of the labels.

FewMedical-XJAU adopts a hierarchical nomenclature system for data annotation (see the fifth column of Figure 3), with the annotation sources and design details elaborated on in Section 3. To further demonstrate the high intra-class variability of FewMedical-XJAU, we select six quantitative metrics to measure image differences—average euclidean distance (Avg Euclidean), average cosine similarity (Avg Cosine), average variance (Avg Variance), average structural similarity index (Avg SSIM), average peak signal-to-noise ratio (Avg PSNR), and average color standard deviation (Avg Color Std Dev)—and conduct a comparative analysis against nine other mainstream publicly available plant leaf datasets.

**Figure 4:**
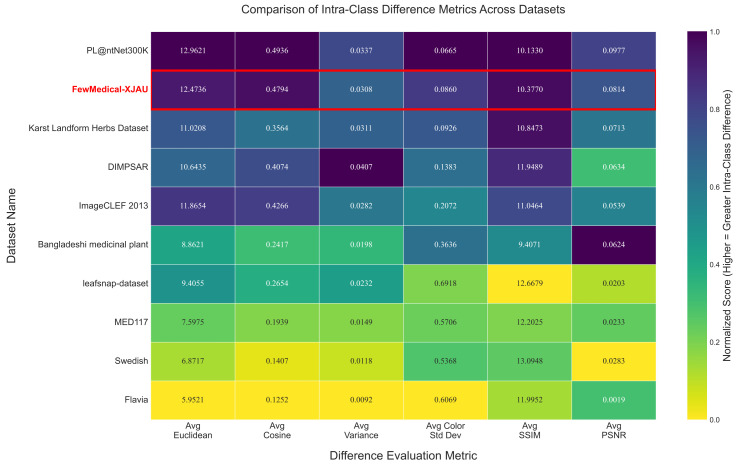
Comparison of statistical characteristics across different image datasets. The metrics are defined as follows: Avg Euclidean and Avg Cosine measure feature dissimilarity; Avg Variance reflects image detail and contrast; Avg SSIM assesses perceptual similarity; Avg PSNR evaluates image quality (higher values indicate better quality); and Avg Color Std Dev indicates color richness. Higher values of Avg Euclidean and Avg Cosine directly correspond to greater intra-class variability. Conversely, lower values of Avg SSIM and Avg PSNR imply higher intra-class variability. Additionally, larger Avg Variance and Avg Color Std Dev typically correspond to increased image complexity and color diversity, which often correlate with greater intra-class variability.

To eliminate the influence of differing metric scales, we first normalize all six indicators and then compute an overall dissimilarity score for each dataset, which is used to rank the datasets accordingly. The comparative results are visualized in the heatmap shown in Figure 4, where the color intensity represents the normalized dissimilarity score—darker colors indicate greater intra-class variability. The numeric values inside each cell correspond to the raw metric results. All datasets are ordered from highest to lowest based on their overall normalized dissimilarity scores (the mean of all normalized metrics). As shown in Figure 4, FewMedical-XJAU consistently ranks among the top datasets in terms of intra-class variability, especially excelling in average cosine similarity (reflecting deep feature differences) and average color standard deviation (capturing color variation). The red box highlights FewMedical-XJAU, underscoring its superior comprehensive ranking and metric performance compared to nine other publicly available plant datasets. This clearly demonstrates that our constructed dataset exhibits significant intra-class diversity, providing a high-quality data foundation for training more robust deep learning models.

### 2.3. Few-Shot Learning

#### 2.3.1. Transfer Learning-Based Few-Shot Methods

Transfer learning [18] has emerged as a highly effective paradigm for addressing the challenges of few-shot image classification. Its fundamental principle lies in transferring knowledge acquired from a source domain to a target domain, thereby enhancing model performance in scenarios with limited labeled data. Specifically, transfer learning seeks to leverage representations or parameters learned from one domain or task and adapt them to a different, yet related, domain or task. This strategy not only reduces the demand for extensive labeled datasets but also enables deep learning models to generalize effectively in data-scarce environments. The standard workflow typically involves pretraining a model on a large-scale dataset (e.g., ImageNet), followed by fine-tuning on the target domain with limited supervision.

To address the distribution shift between domains, Wang et al. (2017) [19] proposed the Balanced Distribution Adaptation (BDA) algorithm, which introduces a balancing factor to adaptively regulate the attention between marginal and conditional distribution alignment. This facilitates more effective domain transfer and improves classification performance in the target domain. Along similar lines, Liu et al. (2020) [20] incorporated attention mechanisms to reweight and recombine domain-specific feature representations, demonstrating competitive performance in few-shot classification settings.

Despite their effectiveness, transfer learning-based approaches face significant challenges when there exists a substantial domain discrepancy. Under such conditions, the efficiency of knowledge transfer diminishes. Critical issues include how to construct robust and discriminative representations for the target domain and how to establish reliable mappings between source and target domain data and labels. These remain open problems in the field of few-shot learning.

#### 2.3.2. Meta-Learning-Based Few-Shot Methods

Meta-learning, also known as “learning to learn,” is a general learning paradigm aimed at enhancing a model’s ability to rapidly adapt to new tasks. Its core idea is to enable the model to accumulate effective prior knowledge by training across multiple related tasks, thereby improving its generalization capability to unseen tasks. Meta-learning typically employs a task-driven training mechanism to achieve efficient adaptation to novel tasks.

Currently, meta-learning methods can be broadly categorized into two main types:

Optimization-based meta-learning: These methods focus on learning a universal model initialization such that the model can quickly achieve optimal performance on new tasks with only a few gradient updates. A representative example is Model-Agnostic Meta-Learning (MAML) proposed by Finn et al. (2017) [21], which determines a global optimization direction by computing gradients for each task. Building upon this, various improvements have been proposed, such as MAML++ [22] which enhances flexibility and computational efficiency; Task-Agnostic Meta-Learning (TAML) which alleviates computational bottlenecks caused by higher-order derivatives; and Implicit MAML (IMAML) which simplifies gradient computation through novel loss function designs.

Metric-based meta-learning: The core of these methods lies in designing effective similarity metrics to measure the distance between query samples and support samples, facilitating few-shot classification. Prototypical Networks (Snell et al., 2017) [23] compute the mean feature vector of support samples for each class as a prototype and classify query samples based on their Euclidean distance to these prototypes. Bertinetto et al. (2018) [24] introduced an attention mechanism to dynamically predict class network parameters for the target task, improving classification accuracy. Cao and Zhang (2022) [25] enhanced feature matching robustness by introducing a semantic alignment loss that optimizes the alignment of two self-similarity matrices.

Although these methods have demonstrated excellent performance in general image classification tasks, they often emphasize global image feature representations. Consequently, in fine-grained classification tasks—especially when intra-class variance is high and inter-class variance is low—their ability to capture subtle local features remains insufficient, limiting classification performance.

### 2.4. CLIP Model

CLIP (Contrastive Language–Image Pretraining) [15] is a multimodal pretraining model proposed by OpenAI, designed to map images and text into a unified semantic space to achieve cross-modal understanding and retrieval. Its core idea is to perform contrastive learning on large-scale image–text pairs, such that the features of images and their corresponding textual descriptions are closely aligned in the feature space. This approach endows the model with strong generalization capabilities, particularly excelling in zero-shot learning scenarios.

In computer vision, zero-shot learning originally refers to the ability of a model to generalize to unseen object categories (Lampert et al., 2009) [26]. CLIP [15] extends this concept by enhancing the model’s generalization ability across entirely new datasets and applying it to joint visual and linguistic modeling.

CLIP consists of two core encoders:

Image encoder: typically adopts Vision Transformer (ViT) [27] or ResNet [28] architectures to extract image feature representations;

Text encoder: based on the Transformer [29] architecture, it maps textual descriptions into a semantic space aligned with image features.

During pretraining, CLIP [15] learns to determine whether an image–text pair matches by optimizing a contrastive loss. In zero-shot classification tasks, the model inputs each class name (often combined with prompt text) into the text encoder to generate class features. These are then compared with the image features via cosine similarity, followed by temperature scaling and softmax normalization to yield class probabilities. This mechanism can be viewed as the image encoder acting as a visual backbone network, while the text encoder functions as a “hypernetwork” (Ha et al., 2016) [30], dynamically generating classifier weights based on class descriptions. This design principle traces back to the works of Lei Ba et al. (2015) [31] and Elhoseiny et al. (2013) [32].

However, CLIP [15]’s practical performance can be affected by label ambiguity and the lack of contextual information. To mitigate these issues, prompt engineering techniques are widely employed. For example, using the default prompt template “A photo of a label” in ImageNet classification tasks can improve accuracy by approximately 1.3%.

### 2.5. Deep Learning Architectures for Plant Image Classification

With the rise of convolutional neural networks (CNNs), feature extraction for plant images has shifted from handcrafted methods to automated deep learning approaches. CNNs can capture leaf structures, textures, and vein patterns at multiple scales and levels, significantly improving classification accuracy. Early studies primarily focused on combining global and local features and optimizing model architectures to balance performance and computational cost. This section reviews various deep learning architectures and methods applied to plant classification.

Lee et al. (2017) [33] proposed a hybrid model combining global and local features by training a CNN on whole-leaf images and using a deconvolutional network to extract salient features, integrating local details such as vein patterns. While effective in species discrimination, this approach requires training two CNNs, resulting in high computational overhead. Wei Tan et al. (2018) [34] designed D-leaf, a simpler and more computationally efficient model consisting of three convolutional and fully connected layers. Dudi and Rajesh (2022) [35] introduced the Shark Smell Whale Optimization Algorithm (SS-WOA) to optimize CNN parameters, accelerating convergence and enhancing classification accuracy. Quach et al. (2023) [36] proposed a hybrid method combining handcrafted features (e.g., color, vein, shape) with CNN features, which performs well on small datasets but shows limitations on large-scale data. Tang et al. (2025) [14] addressed the challenge of complex backgrounds in medicinal plant recognition by proposing MPR-net, which employs a dual-branch attention module to fuse window-based self-attention with global tokens, enhancing local–global feature interaction and improving recognition and generalization.

With the widespread adoption of Transformer architectures in computer vision, Vision Transformer (ViT) has demonstrated strong potential for handling large-scale and complex plant classification tasks. Hoang et al. (2023) [37] incorporated a hierarchical loss function into the ViT-B/16 model, jointly considering errors at species, genus, and family levels to improve robustness in hierarchical classification. Fang et al. (2024) [38] proposed CSDNet, which combines contrastive learning and self-distillation, using content-aware convolutional kernels to convert high-level semantics into low-level details, storing historical sample features in a dynamic memory queue, and applying self-distillation during inference to enhance discrimination of similar classes while reducing memory cost. Arkel Rios et al. (2024) [39] addressed attention collapse in frozen ViTs by introducing an Intermediate Layer Adapter (ILA), which inserts a dual-branch spatial downsampling module between Transformer layers to enrich feature hierarchies and attention diversity, integrating botanical taxonomy information to improve model robustness and classification performance.

In tasks involving rare or endangered plants with extremely limited samples, few-shot and zero-shot learning have become research hotspots. Zou et al. (2023) [40] proposed S^2^CL-LeafNet, which enhances key attribute representation such as veins and shapes through multi-layer CNN features and attention fusion, improving classification performance. Zulfiqar and Izquierdo (2025) [17] introduced a local selective attention mechanism to generate highly discriminative plant features, which combined with feature reconstruction further improves classification accuracy.

Table 2 summarizes the performance of the aforementioned plant classifiers on their respective datasets.

## 3. Proposed Dataset

This section focuses on the collection, processing, and feature description of FewMedical-XJAU. The collected images not only include complex backgrounds but also cover different seasons, various plant organs, and multiple shooting perspectives. Data annotation follows a standardized classification labeling method and adheres to the international binomial nomenclature for naming.

### 3.1. Data Collection and Processing

Since 2023, our research team has conducted systematic image collection of medicinal plants in representative ecological regions, including the Altai and Tianshan Mountains. Image acquisition was primarily performed using a high-resolution Canon 90D camera and a Huawei Mate50 smartphone, following standardized recording procedures. To compensate for species with insufficient image data, a portion of the dataset was supplemented with synthetically generated images. Ultimately, 86% of the images were obtained through direct photography, while 14% were synthetically generated.

**Figure 5:**
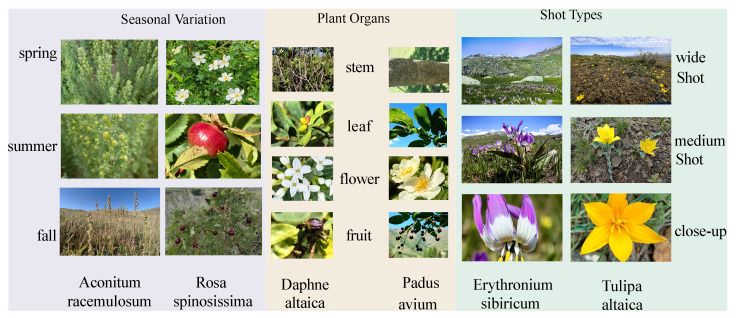
The figure presents typical examples of plants under different seasonal conditions, plant organs, and photography types. On the left, Seasonal Variation shows the morphological changes of the same plant across different seasons. In the middle, Plant Organs displays the visual differences between various organs of the same plant. On the right, Shot Types illustrates how the same plant appears under different photographic angles.

As illustrated on the left side of Figure 5 under “Seasonal Variation” the dataset spans the spring, summer, and fall to systematically capture the morphological characteristics of plants at various growth stages. It further encompasses multiple plant organs—including roots, stems, leaves, flowers, and fruits—as shown in the middle section labeled “Plant Organs.” To provide comprehensive visual coverage, three shot scales (wide, medium, and close-up) are employed, enabling the documentation of both global plant structures and fine-grained local details, as depicted in the right section under “Shot Types”.

**Figure 6:**
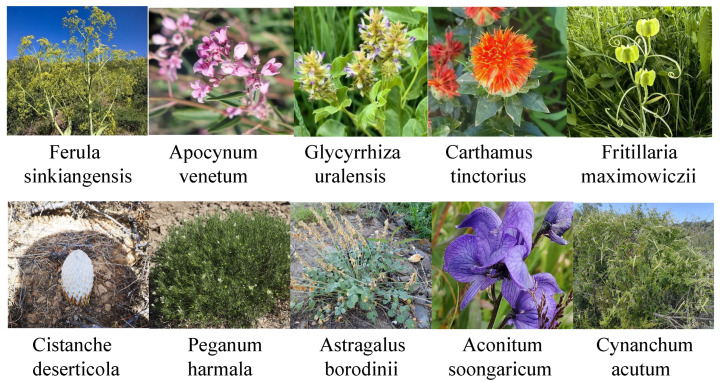
Representative medicinal plant images exhibiting distinctive characteristics of Xinjiang.

During the data collection process, special attention was given to rare medicinal plants with distinct regional characteristics of Xinjiang. As illustrated in Figure 6, several representative herbaceous species endemic to the Xinjiang region—such as *Apocynum venetum*, *Carthamus tinctorius*, *Ferula sinkiangensis*, *Glycyrrhiza uralensis*, and *Fritillaria maximowiczii*—are documented. These species hold significant medicinal value and play an important role in traditional Chinese medicine.

After acquiring the medicinal plant images, the raw data were systematically organized based on the principles of plant taxonomy and stored in a hierarchically structured directory with clearly defined classifications. Following authoritative taxonomic standards outlined in Flora of China [41] and Medicinal Plant Taxonomy [42], a three-tier classification framework—Phylum–Family–Genus species—was adopted. Specifically, each phylum-level folder contains multiple subfolders corresponding to plant families, and within each family folder, further subdirectories are created for individual species, ensuring that each species is allocated an independent storage space.

Image file names follow a standardized taxonomic annotation format: *Phylum_Family_ Genus species*. In this naming convention, *Phylum* denotes the plant division, *Family* indicates the corresponding plant family, and *Genus species* represents the scientific name in accordance with Linnaean binomial nomenclature, which consists of the genus name (Genus) followed by the species epithet (Species) [43].

### 3.2. Data Description

**Figure 7:**
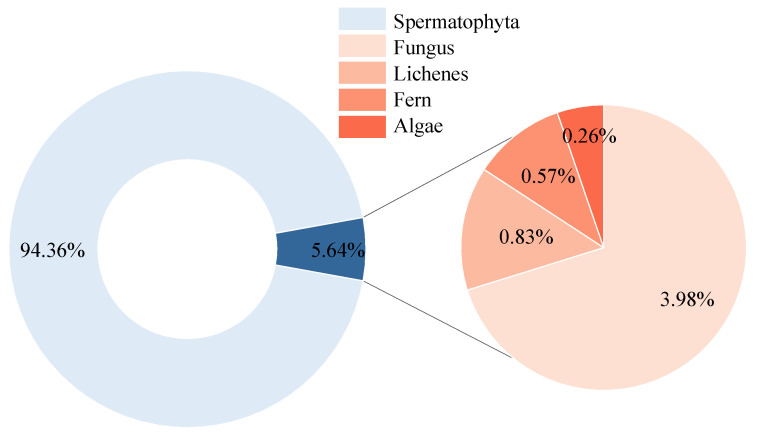
The proportional distribution of five plant phyla in FewMedical-XJAU is illustrated in the form of a dual-ring chart. The outer ring represents the overall distribution across all phyla, with Spermatophyta accounting for a dominant 94.36%. The inner ring provides a detailed breakdown of the non-seed plant phyla: Fungus accounts for 3.98%, Lichenes for 0.83%, Fern for 0.57%, and Algae comprises the smallest proportion, at only 0.26%.

The FewMedical-XJAU dataset comprises 5 phyla, 125 families, and 540 plant species, totaling 4992 images. Figure 7 illustrates the proportional distribution of these phyla: Spermatophyta accounts for 94.36%, Fungus 3.98%, Lichenes 0.83%, Fern 0.57%, and Algae the least at 0.26%. The proportion of Spermatophyta exceeds the combined total of the other phyla by approximately 20 times.

**Figure 8:**
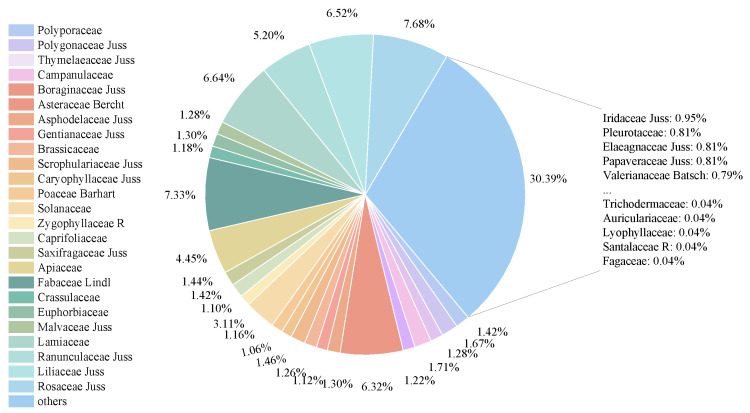
The proportion of data by family classification in FewMedical-XJAU shows that the Rosaceae Juss accounts for the largest proportion, at 7.33%. Families with a proportion of less than 1% are categorized as “Others,” which together account for 30.39%. The right side of the chart lists several families with a proportion of less than 1% and their corresponding percentages.

Figure 8 illustrates the distribution of image proportions across different plant families within the dataset. The top five families with the highest representation are Rosaceae Juss (7.68%), Fabaceae Lindl(7.33%), Lamiaceae (6.64%), Liliaceae Juss (6.52%), and Asteraceae Bercht (6.32%). Families with proportions below 1% were grouped under the category “Others.” On the right side of the pie chart, the five most and least represented families within this “Others” category are listed. Among them, Trichodermaceae, Auriculariaceae, Lyophyllaceae, Santalaceae R, and Fagaceae are the least represented families in the entire dataset, each accounting for only 0.04% of the total images.

In summary, Figure 7 and Figure 8 clearly demonstrate that the FewMedical-XJAU dataset exhibits the characteristics of a typical small-sample distribution, with a highly imbalanced number of images across categories. This highlights the real-world challenge of severe data scarcity for rare medicinal plants in training samples. Such scarcity is particularly difficult for conventional deep learning methods to address, as they generally rely on large-scale and relatively balanced datasets to achieve robust performance. Therefore, few-shot learning is especially necessary and applicable in this domain, as it is specifically designed to learn discriminative features and achieve effective generalization from only a few samples. By providing a benchmark that realistically reflects the data distribution of rare medicinal plants, the FewMedical-XJAU dataset offers a critical resource to advance research on few-shot learning methods for medicinal plant classification.

## 4. Proposed Method

This chapter presents a few-shot image classification framework enhanced by cross-modal visual–textual integration. The capabilities of DeepBDC [44] in capturing intra-class distributions through covariance modeling, and the effectiveness of CLIP [15] in leveraging textual priors for fine-grained category discrimination, are studied. To address the challenges of large intra-class variance and subtle inter-class differences among plant species, a multimodal fusion strategy is incorporated within a deep metric learning framework.

To further enhance category representation and improve cross-modal generalization, a Class-Aware Structured Text Prompt Construction is proposed. This approach encodes rich semantic information—such as appearance characteristics, growth habits, and functional features—into the textual modality.

Finally, a task-adaptive fusion mechanism is introduced to dynamically weight each modality based on support set performance, thereby improving classification robustness under limited data conditions.

**Figure 9:**
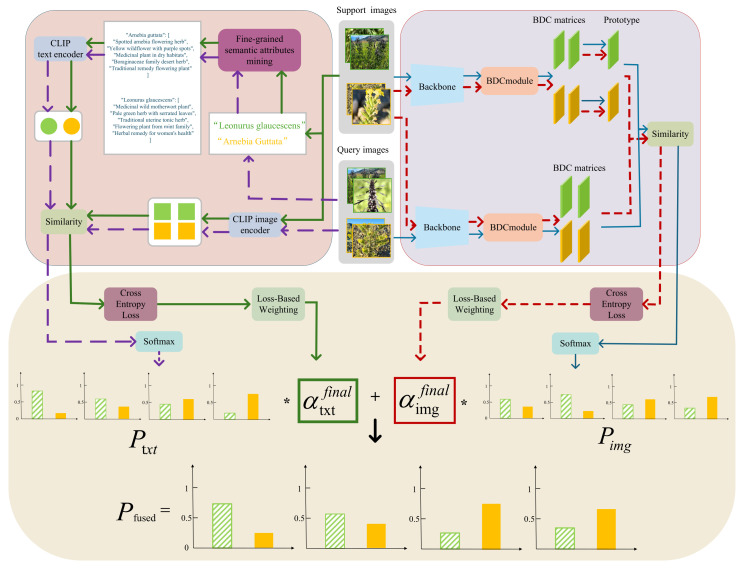
The complete model framework is illustrated. The red arrows indicate the dynamic computation of weights for the visual branch based on the DeepBDC [44] model using the support set, while the green arrows indicate the dynamic computation of weights for the textual branch based on the CLIP [15] model using the support set. The purple arrows denote the prediction probabilities obtained by feeding the query set and class-aware structured text into the CLIP [15] model, and the blue arrows represent the prediction probabilities generated from the query set and support prototypes via the DeepBDC [44] model. Each branch has four outputs, corresponding to four query images. Each bar chart shows the predicted probabilities of the query image over two classes, with bars colored yellow and green to distinguish the classes. To improve visibility in black-and-white prints, the green bars use diagonal line hatching, while the yellow bars remain solid. Finally, the outputs from both branches are adaptively fused using a dynamic weighting strategy to produce a more robust prediction.

As illustrated in Figure 9, the top-left box illustrates the use of the CLIP [15] model to align visual information with class-aware structured text, producing a probability distribution representing the prediction from the text branch. The top-right box shows a second probability distribution generated by metric learning, representing the prediction from the pure visual branch. Finally, a dynamic fusion mechanism is applied to combine the two probability distributions, yielding the final prediction result.

### 4.1. Multimodal Feature Fusion Strategy

DeepBDC [44] is a parameter-free spatial pooling layer designed to extract second-order statistical representations of images, thereby providing a more precise characterization of intra-class feature distributions. Given an input image *z*, a parameterized network $f_{\theta }$ maps it to a feature tensor of shape h×w×d, where *h* and *w* are the spatial dimensions, and *d* is the number of channels. This tensor is then reshaped into a matrix $X\in R^{(h\cdot w)\times d}$, where each row corresponds to a feature vector from a spatial location.

The Brownian Distance Covariance (BDC) representation of the image is computed through the following steps. First, a Euclidean distance matrix $\hat{A}$ is computed, where each element ${\hat{A}}_{kl}$ represents the distance between feature vectors $x_{k}$ and $x_{l}$:

(1) $${\hat{A}}_{kl}=∥x_{k}-x_{l}∥$$

Double-center the distance matrix $\hat{A}$ to obtain the final BDC matrix *A* (the “BDC matrices” shown in Figure 9):

(2) $$A_{kl}={\hat{A}}_{kl}-\frac{1}{d}\sum_{i=1}^{d}{\hat{A}}_{il}-\frac{1}{d}\sum_{j=1}^{d}{\hat{A}}_{kj}+\frac{1}{d^{2}}\sum_{i=1}^{d}\sum_{j=1}^{d}{\hat{A}}_{ij}$$

In our method (as illustrated in Figure 9), for each class in the support set (e.g., the “green” and “yellow” classes), we first generate a BDC matrix for every support sample using the backbone network and the DeepBDC module. Then, by averaging the BDC matrices of all support samples belonging to the same class element-wise, we obtain a compact class prototype matrix $P_{c}$ (the “prototype” shown in Figure 9), which serves as the representative of that class.

The similarity between the query image’s BDC matrix $A_{\mathrm{query}}$ and each class prototype $P_{c}$ is computed using the matrix trace operation, as defined in the original DeepBDC paper [44]:

(3) $$\rho \left(A_{\mathrm{query}},P_{c}\right)=\mathrm{tr}\left(A_{\mathrm{query}}^{⊤}P_{c}\right)$$

This measures the dependency between the two distributions represented by the BDC matrices. Finally, the query is assigned to the class with the highest similarity score, enabling efficient and accurate classification with strong discriminative power and generalization capability under few-shot learning settings.

However, in scenarios involving visually similar yet semantically distinct categories, the model may suffer from misclassification. This limitation stems primarily from the exclusive reliance on visual modality, which lacks the incorporation of semantic priors related to class identity.

To compensate for the limitations of the image modality in semantic understanding, this study introduces the CLIP [15] (Contrastive Language–Image Pretraining) model. CLIP [15] leverages semantic prior knowledge from natural language prompts to construct a joint vision–language feature space.

For instance, by constructing prompts such as “a picture of a [plant species]”, CLIP [15] can generate category embeddings that encapsulate relevant semantic information. These vectors serve as complementary representations to the class labels and help the model perform semantically guided differentiation of visual features.

Building upon this, a unified representation method, BDCC (Bilinear Deep Cross-modal Composition), is proposed to integrate image features extracted by DeepBDC [44] and text features encoded by CLIP [15] within a deep metric learning framework. Both modalities are first projected into a shared embedding space to achieve semantic alignment, followed by an efficient fusion mechanism that jointly models visual and textual information, effectively leveraging their complementary strengths.

### 4.2. Class-Aware Structured Text Prompt Construction

**Figure 10:**
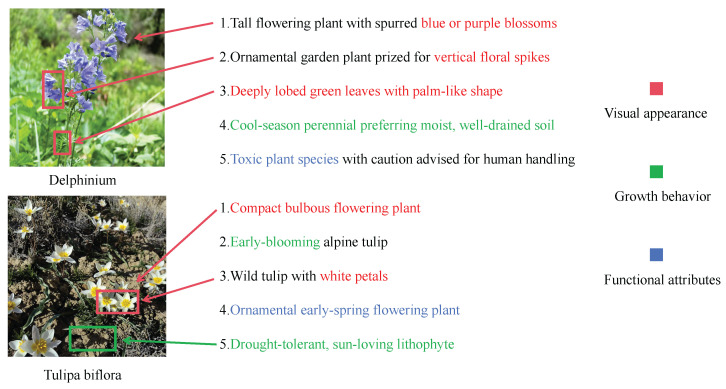
The figure presents a Class-Aware Structured Text Prompt Construction strategy. For each category, five semantically relevant textual descriptions are designed, covering multiple semantic dimensions including visual appearance (marked in red), growth behavior (marked in green), and functional attributes (marked in blue).

To enhance the model’s understanding of category-level semantics, we propose a Class-Aware Structured Text Prompt Construction strategy to replace the conventional single-template or prompt-ensemble methods typically used in CLIP [15]. Specifically, as illustrated in Figure 9, the Fine-grained Semantic Attributes Mining leverages GPT-4.1 mini to generate multiple semantically structured textual descriptions for each category, covering diverse aspects such as visual appearance, functional usage, and growth behavior. These generated descriptions are carefully reviewed to ensure correctness and consistency. Finally, for each category, five semantically relevant textual prompts are designed and stored in JSON format, as shown in Figure 10.

Unlike the standard CLIP [15] prompt templates such as a photo of a class_name or This is a photo of a class_name, the five category-specific descriptions are concatenated using English commas to generate a semantically rich compound sentence. For example, for the category *Delphinium*, the resulting prompt is:

*“Tall flowering plant with spurred blue or purple blossoms, ornamental garden plant prized for vertical floral spikes, deeply lobed green leaves with palm-like shape, cool-season perennial preferring moist, well-drained soil, toxic plant species with caution advised for human handling.”*

This strategy effectively compresses and integrates category-related semantic information, enabling each prompt to convey not only the basic class name but also rich contextual and inter-class discriminative features [45].

The implementation process is carried out by the text feature preparation module, which involves the following steps:

1. Reading multiple textual descriptions for each category from a predefined JSON file.
2. Concatenating these descriptions into a compound sentence using English commas.
3. Encoding the concatenated text using a pretrained language model to generate semantic embedding vectors.

### 4.3. Task-Adaptive Fusion Strategy Based on Support Set Performance with Weight Smoothing

#### 4.3.1. Dynamic Weight Computation

For each test task (episode), the predictions of DeepBDC [44] and CLIP [15] are first obtained on the support set, and their cross-entropy losses with respect to the ground-truth labels are computed, denoted as $L_{img}$ and $L_{txt}$, respectively. To adaptively reflect the relative reliability of the two modalities in the current task, the raw weights are calculated as the inverse of the corresponding losses:

(4) $$w_{img}=\frac{1}{L_{img}+\epsilon },\;w_{txt}=\frac{1}{L_{txt}+\epsilon },$$

where ϵ is a small constant to prevent division by zero. The weights are then normalized to obtain the fusion coefficients:

(5) $$\alpha_{img}=\frac{w_{img}}{w_{img}+w_{txt}},\;\alpha_{txt}=\frac{w_{txt}}{w_{img}+w_{txt}}.$$

To avoid excessive bias toward a single modality, $\alpha_{img}$ and $\alpha_{txt}$ are smoothed, improving stability across tasks. It is important to emphasize that these weights are not fixed during training; rather, they are computed in real time for each test task based on support set performance and used to fuse the query set predictions. This per-task dynamic weighting strategy follows the concept of dynamic multimodal decision weighting in few-shot learning [46,47], ensuring that the final prediction relies more on the modality that performs better on the current task’s support set, thereby enabling efficient and adaptive multi-modal fusion.

Furthermore, the method supports unimodal operation: when text prompts are unavailable, $\alpha_{txt}=0$ and only the image modality is used; conversely, when image data is missing or of poor quality, $\alpha_{img}=0$ and the model operates solely based on the text modality, enhancing practical applicability and robustness under varying modality availability.

In the bottom panel of Figure 9, solid green lines indicate that support set images are processed through the CLIP [15] image encoder, while their corresponding species names are converted into textual descriptions via Fine-grained Semantic Attributes Mining (see Section 4.2). These textual descriptions are stored as JSON files and encoded using the CLIP [15] text encoder, with the green arrow representing the resulting BDCC text-branch weights for the support set. Red dashed lines indicate the BDCC image-branch weight computation; purple dashed lines denote the query set predictions from the BDCC text branch; and blue arrows indicate the BDCC image-branch predictions. Finally, the weights from both branches are applied to their respective predictions and fused into the final output, as indicated by the black arrow in the figure.

#### 4.3.2. Weight Smoothing Mechanism

In practical tasks, the limited size of the support set may lead to significant fluctuations in loss values due to data variance, potentially causing extreme bias in the fusion weights toward a single modality and compromising overall prediction stability. To mitigate this, a weight smoothing mechanism is introduced, which linearly interpolates the dynamic fusion weights as follows:

(6) $$\alpha_{\mathrm{img}}^{\mathrm{final}}=\left(1-\beta \right)\alpha_{\mathrm{img}}+\beta \cdot 0.5,\;\alpha_{\mathrm{txt}}^{\mathrm{final}}=\left(1-\beta \right)\alpha_{\mathrm{txt}}+\beta \cdot 0.5,$$

where β∈[0,1] is the smoothing factor (set to 0.5 in this work). When β=0, the fusion is fully dynamic; when β=1, the fusion reduces to an equal average. This mechanism effectively mitigates the instability caused by extreme weight allocations.

#### 4.3.3. Fusion Prediction and Classification

With the smoothed weights obtained, the predictions of DeepBDC [44] and CLIP [15] on the query set are converted to probabilities by applying softmax, and then fused by weighted summation:

(7) $$P_{\mathrm{fused}}=\alpha_{\mathrm{img}}^{\mathrm{final}}\cdot P_{\mathrm{img}}+\alpha_{\mathrm{txt}}^{\mathrm{final}}\cdot P_{\mathrm{txt}},$$

where $P_{\mathrm{img}}$ and $P_{\mathrm{txt}}$ represent the class probability distributions predicted by the image and text models on the query set, respectively. The final predicted class corresponds to the category with the highest probability in $P_{\mathrm{fused}}$.

## 5. Experiment

This chapter presents the experimental setup, including conventional and few-shot classification tasks based on the dataset characteristics. It compares the proposed method with existing approaches on the FewMedical-XJAU dataset and conducts ablation studies to assess the effectiveness of the improvement strategies.

### 5.1. General Classification Tasks

#### 5.1.1. Experimental Details

**Figure 11:**
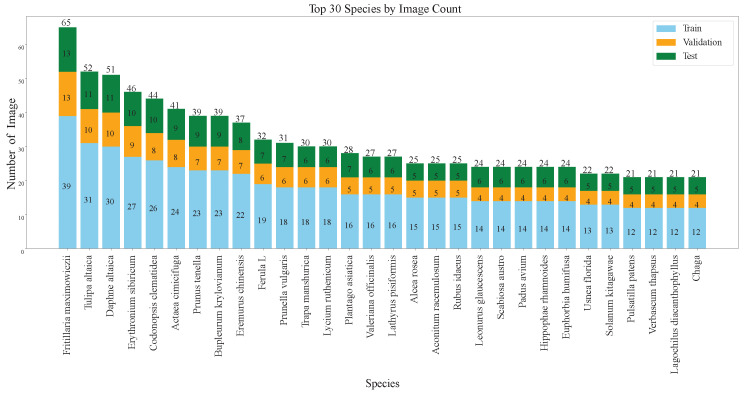
This figure shows the number of images in the training, validation, and test sets for the top 30 most represented plant species in the general classification tasks dataset.

**Figure 12:**
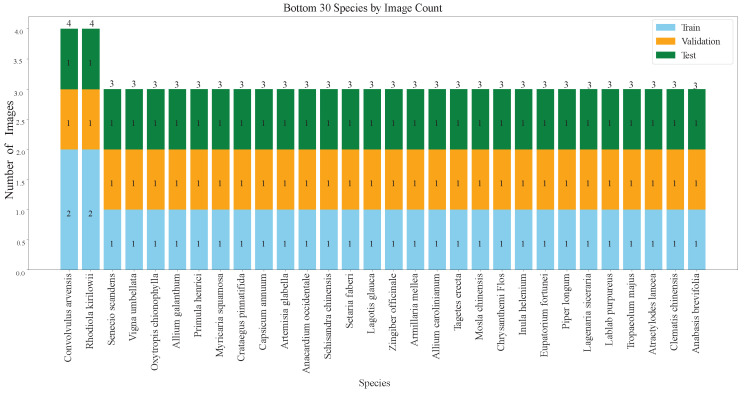
This figure shows the number of images in the training, validation, and test sets for the bottom 30 least represented plant species in the general classification tasks dataset.

In the general classification tasks, considering that each plant species must be included in the training, validation, and test sets, with at least one image in each set, we select species with no fewer than three images to form a subset of the original dataset. This subset comprises 483 plant species, totaling 4804 medicinal plant images. To facilitate model training and testing, we divide this subset into training, validation, and test sets in a 3:1:1 ratio. As shown in Figure 11 and Figure 12, the bar charts visually present the number of images in the training, validation, and test sets for the top 30 and bottom 30 plant species in the general classification tasks dataset.

In terms of model selection, we cover representative convolutional models such as ResNet-50 [28], as well as a series of lightweight models that combine the advantages of CNN and ViT (including MAE [48], MPViT_tiny [49], SwiftFormer_XS [50], SBCFormer_B [51], RepViT-M0.9 [52], and UniRepLKNet–A [53]). All models are trained using the PyTorch 2.5.1 framework on a desktop computer equipped with an NVIDIA GeForce RTX 4090.

To fully utilize computational resources, the batch size is set to powers of two (e.g., 64, 128, 256, 512), thereby maximizing GPU memory usage (24,564 MiB) while avoiding overflow and ensuring training efficiency. The backbone networks of all models are pretrained on the ImageNet-1K [54] dataset, with pretrained weights loaded during training. Specifically, the weights of the fully connected layers are removed and reinitialized, while the remaining layers retain the pretrained parameters. Furthermore, to ensure a fair comparison and maintain consistency with the original methods, each model adopts the optimizer and learning rate settings as specified in its corresponding original paper. Table 3 summarizes the detailed configurations and experimental results of these models on the proposed dataset.

#### 5.1.2. Experimental Evaluation

Based on the experimental results, the performance of various models on conventional classification tasks is generally low, indicating that the limited number of training samples has a significant impact on model performance. Among all models, SwiftFormer_XS [50] exhibits the poorest performance, achieving a Top-1 accuracy of only 5.99%. Similarly, the lightweight models RepViT-M0.9 [52] and MPViT_tiny [49], despite incorporating Vision Transformer modules to enhance global modeling capabilities, achieve Top-1 accuracies of only 22.59% and 43.77%, respectively. This underperformance is partly due to the limited robustness of lightweight architectures in handling complex textures, detailed structures, and background interference. More importantly, the small number of training samples severely restricts the models’ ability to effectively learn discriminative features in fine-grained plant images.

In contrast, representative classic models such as ResNet-50 [28] and MAE [48] also fail to achieve the expected performance in this task. ResNet-50 [28], a pure CNN architecture, attains a Top-1 accuracy of 35.20%, indicating certain limitations in capturing multi-scale features and fine textures of plant images. On the other hand, MAE [48], although based on Vision Transformer and possessing strong representational power, heavily relies on large-scale datasets and masking strategies during training, resulting in a Top-1 accuracy of only 39.71%. SBCFormer_B [51], by introducing a structural perception module, demonstrates advantages in modeling local geometric features of plants, achieving a Top-1 accuracy of 57.79%. Nevertheless, the overall performance of these models remains suboptimal, primarily due to the limited number of training samples, which constrains their transferability and generalization capabilities. In addition, all models are fine-tuned from pretrained weights on ImageNet-1K [54], a dataset composed predominantly of general natural images. The substantial domain shift between ImageNet-1K [54] and the FewMedical-XJAU dataset, in terms of both content and structure, results in a lack of prior perception of plant morphological features, further limiting model performance.

Among all evaluated models, UniRepLKNet-A [53] demonstrates the strongest performance, achieving a Top-1 accuracy of 74.61%. By incorporating large receptive field convolutions and a unified feature representation mechanism, UniRepLKNet-A [53] enhances its capacity to perceive and model complex plant structural features to a certain extent.

In summary, all models perform below the ideal level on the FewMedical-XJAU dataset. This phenomenon can be primarily attributed to the inherent challenges of the dataset. First, the scarcity of rare medicinal plants results in a limited number of image samples, which is the most critical factor. Second, the dataset predominantly contains rare medicinal plants specific to the Xinjiang region, leading to a lack of domain-specific learning and prior knowledge in the models. Finally, the images exhibit complex backgrounds, large intra-class variance, and high inter-class similarity, further increasing the difficulty of the classification task.

For specific plant recognition tasks, the implementations and code of most related models remain unpublished, making it difficult to reproduce or directly compare them in the present study. Among the few publicly available models, we select MPR-net [14] as a representative example. This model is specifically designed for medicinal plant recognition and classification and incorporates a dual-branch fused attention module to enhance discriminative feature extraction, thereby improving its suitability for medicinal plant identification tasks. In conventional classification tasks, MPR-net [14] demonstrates relatively strong performance, achieving a Top-1 accuracy of 68.75% on our dataset. This performance is primarily attributed to the similarity between its pretraining tasks and some categories in our dataset, which provides the model with prior knowledge and improves classification outcomes. Nevertheless, its overall performance does not reach the level of the best conventional classification models, as it heavily relies on large-scale pretraining. For the numerous rare medicinal plant categories in our dataset, the designed attention mechanism has limited effectiveness, thereby constraining the model’s performance on these categories.

### 5.2. Few-Shot Classification Task

#### 5.2.1. Experimental Details

The dataset for the few-shot classification task is constructed from two subsets of the original dataset, corresponding to the 1-shot and 5-shot experiments. This design ensures that classes with fewer than five images also participate in the classification task, while avoiding the use of data augmentation techniques.

The classes are first randomly partitioned into training, validation, and test sets in a 3:1:1 ratio, with no overlap of classes across the three subsets. For the 1-shot task, training selects classes from the training set that contain at least three images. Specifically, the 1-shot task requires at least two images, with one used as the support set and one as the query set. However, experimental results across multiple datasets show that few-shot methods generally fail to learn effectively when the query set contains only a single image. In 5-way classification, the accuracy remains around 20% (i.e., the probability of random guessing at 1/5) due to the lack of discriminative features. Therefore, the query set size is set to two in order to obtain more reliable and distinguishable results. Similarly, during evaluation, both the support and query sets are drawn from the test set.

The 5-shot setting requires more images per class; thus, only classes with at least seven images are included. Detailed data splits are provided in Table 4.

All models are trained using PyTorch on a machine equipped with an NVIDIA GeForce RTX 4060 GPU. The entire models are fine-tuned for 100 epochs using default optimizers, following the optimizer and learning rate settings specified in the original papers. Official pretrained weights trained on large-scale datasets are utilized for transfer learning, aiming to achieve improved performance and generalization.

#### 5.2.2. Comparative Experiments

This study compares five representative few-shot classification methods: the metric learning method based on prototypical networks (ProtoNet) [23]; the classification method combining a dual-branch structure with deep pooling features (DeepBDC) [44]; a novel transductive few-shot classification approach for CLIP (EM-Dirichlet) [55]; a fine-grained few-shot classification method based on a self-reconstruction mechanism—Self-Reconstruction Network (SRN) [56]; and the fine-grained few-shot classification network integrating multi-level feature reconstruction with attention region enhancement (HMDRN) [57]. All these methods are implemented based on the publicly available code and pretrained parameters provided by the original authors. Experimental results are presented in Table 5.

The experimental results demonstrate that the proposed framework achieves the best performance under both 5-way 1-shot and 5-way 5-shot settings, significantly outperforming all comparison methods. The classical metric learning method ProtoNet [23] constructs class prototypes via feature averaging, but this first-order statistic fails to adequately capture the substantial intra-class variability in the dataset. In contrast, DeepBDC [44] employs second-order statistics, specifically covariance matrices, for category modeling. By capturing correlations between features, it offers a more nuanced representation of class distributions and demonstrates notable advantages in addressing substantial intra-class variance.

SRN [56] and HMDRN [57], designed for fine-grained tasks, leverage self-reconstruction mechanisms and hierarchical attention enhancement, respectively, to guide the model toward discriminative object regions and suppress background interference. While this strategy improves performance over ProtoNet [23], both SRN [56] and HMDRN [57] lack global modeling of intra-class structures compared to DeepBDC [44], resulting in relatively weaker performance in fine-grained scenarios characterized by subtle inter-class differences and significant intra-class variations.

EM-Dirichlet [55] relies on generic, templated textual prompts that lack the fine-grained semantic information necessary to distinguish highly similar species. Consequently, it fails to fully exploit the potential of the textual modality and cannot effectively address the core challenge of low inter-class variance.

Compared to existing approaches, BDCC exhibits superior capability in addressing the challenges of high intra-class variance and low inter-class variance in fine-grained classification tasks. First, in contrast to ProtoNet [23], which constructs class prototypes using only first-order statistics, BDCC incorporates the second-order statistical modeling of DeepBDC [44], enabling more effective representation of intra-class structure and inter-feature correlations, thereby enhancing robustness to complex sample distributions.

Second, although SRN [56] and HMDRN [57] improve focus on discriminative regions through self-reconstruction and multi-level attention mechanisms, their capacity to capture global intra-class structure remains limited. BDCC addresses this by integrating CLIP-based semantic priors and introducing a Class-Aware Structured Text Prompt Construction strategy, which overcomes the limitations of purely visual attention and significantly enhances the ability to distinguish between visually similar categories.

Finally, unlike EM-Dirichlet [55], which relies on generic, template-based prompts lacking fine-grained semantic precision, BDCC employs multidimensional semantic guidance and an adaptive dynamic fusion mechanism to ensure robust and context-sensitive decision making across diverse sample conditions. Overall, BDCC systematically addresses the limitations of both unimodal and conventional multimodal methods, demonstrating enhanced adaptability and performance in challenging fine-grained classification scenarios.

#### 5.2.3. Ablation Experiments

To verify the effectiveness of the proposed design strategies—including the Multimodal Feature Fusion Strategy, the Class-Aware Structured Text Prompt Construction, and the Task-Adaptive Fusion Strategy Based on Support Set Performance with Weight Smoothing—ablation studies are conducted.

As shown in Table 6, when using CLIP [15]’s text features alone, the accuracy with prompt-ensemble methods (“a photo of class”) is 64.93%, whereas adopting Class-Aware Structured Textual Prompts increases the accuracy to 71.35%. This indicates that Class-Aware Structured Text Prompt Construction effectively enriches the semantic representation of text features, provides more discriminative contextual information, enhances the model’s understanding and differentiation of category semantics, and facilitates the capture of subtle inter-class differences, thereby significantly improving accuracy in fine-grained classification tasks.

Furthermore, based on DeepBDC [44] for modeling visual features, text prompts provided by CLIP [15] are incorporated, and a simple posterior probability fusion of the image and text model outputs is performed during inference using a fixed weighting scheme (e.g., 0.5:0.5). This leads to a significant improvement in model performance. In the 1-shot setting, accuracy increases from 79.54% with generic text prompts to 82.32% with Class-Aware Structured Text Prompt Construction; in the 5-shot setting, accuracy improves from 88.13% to 88.94%. Despite the simplicity of the fusion approach, the results demonstrate strong complementarity between visual and textual modalities in few-shot recognition. The relatively smaller gain in the 5-shot scenario suggests that, with sufficient image samples, the model can already learn discriminative class features, thereby diminishing the marginal benefit of textual prompts. This further highlights the importance of structured semantic information in enhancing class-level understanding, especially under low-shot conditions.

To validate the effectiveness of the proposed fusion strategy, experiments are conducted on different fusion methods. The results are presented in Table 7.

First, a simple fixed-weight fusion strategy (with weights of 0.5/0.5) serves as the baseline. While straightforward to implement, it lacks task-adaptability. In contrast, the adaptive weight dynamic fusion strategy based on confidence [46] proves unreliable; its susceptibility to model overconfidence leads to a performance degradation in the 1-shot scenario (from 79.54% to 78.73%), demonstrating that a naive dynamic approach is insufficient.

Second, the adaptive weight dynamic fusion strategy based on support set performance [47] introduces task-level adaptability by dynamically assigning weights according to the performance of each modality on the current task [47]. Compared with the adaptive weight dynamic fusion strategy based on confidence [46], this method achieves a significant improvement in the 5-shot setting (88.53%), but in the 1-shot setting, its overall performance remains comparable to that of the simple fixed-weight fusion strategy. This also reflects a practical challenge in real-world applications: the limited number of samples per class can markedly affect model performance and stability. Insufficient samples hinder the model’s ability to fully capture the intra-class diversity, increasing the risk of underfitting and reducing generalization capacity. Moreover, the discriminative ability between classes is weakened—particularly for visually similar classes—making accurate classification more difficult. In addition, a small sample size often leads to large fluctuations in evaluation metrics, making it challenging to obtain a stable and reliable measure of the model’s true performance.

To address these limitations, we propose a final task-adaptive fusion strategy that builds upon the above methods by introducing a weight smoothing mechanism, which effectively suppresses drastic weight fluctuations under extreme task conditions. This strategy achieves the best performance in both the 1-shot and 5-shot settings, with accuracies of 83.02% and 89.15%, respectively. Experimental results clearly demonstrate that weight smoothing is a key factor in improving the stability and generalization of the fusion strategy in few-shot scenarios.

It is worth emphasizing that when using existing few-shot learning datasets for “unseen queries”—that is, samples from novel classes—the number of support samples per class (n-shot) is typically predetermined to construct class prototypes, with common settings being 1-shot and 5-shot. The specific number of support samples depends on the experimental design and task requirements; however, when the number of available samples for a class is extremely limited (e.g., only two images), a 1-shot setting is generally adopted to ensure that the remaining images can be used as query samples during testing. To enable the CLIP sub-model to accurately recognize new classes, the text descriptions should be rich and cover multiple aspects of the class—such as appearance, function, behavior, and other distinctive characteristics—highlighting unique details and incorporating contextual information. At the same time, the descriptions should remain concise and be expressed through multiple structured prompts to convey the full semantic meaning, thereby enhancing the model’s discriminative capability.

#### 5.2.4. Result Visualization

**Figure 13:**
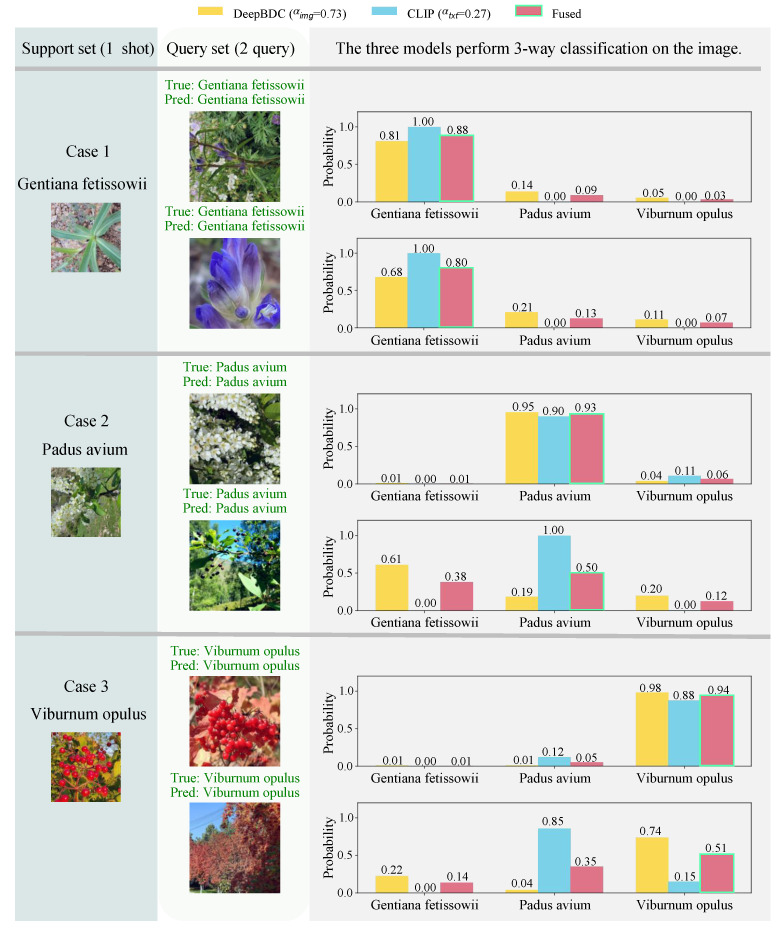
The qualitative visualization of the model’s predictive performance on the query set in a representative 3-way 1-shot task.

Figure 13 provides a qualitative visualization of the model’s predictive performance on the query set within a representative 3-way 1-shot task. For each class, the corresponding support set image is shown on the left, and the query set image for classification is presented in the center. The right side displays class probability histograms for the query sample predicted by DeepBDC [44], CLIP [15], and their fused model. The predicted probability of the ground-truth class is highlighted with a bright green box. Prediction results are annotated above each histogram, with green text indicating correct predictions and red text indicating incorrect ones.

The weights in the legend are computed during test-time inference based on the support set of the current task: the classification losses of DeepBDC [44] and CLIP [15] are first calculated on the support set, and the weights are then set as the inverse of these losses and normalized to reflect the relative predictive reliability of the two models for the given task. The values above the bars represent the mean predicted probability of each class across all query samples. Specifically, for an individual query sample, the sum of predicted probabilities across all classes is 1. For instance, in a three-class task, if a query image has predicted probabilities *a*, *b*, and *c* for classes A, B, and C, respectively, then a+b+c=1.

1. **Case 1:** ***Gentianna fetissowii*****—Synergistic Enhancement via Cross-Modal Consistency**
  This case demonstrates synergistic enhancement under conditions of high cross-modal consistency. The visual features exhibit strong discriminability, and the textual prompts provide clear semantics, resulting in a well-aligned multimodal feature space. Both the visual and textual branches independently produce high-confidence correct predictions. Fusion yields a lower-entropy posterior distribution, reinforcing the decision boundary. This highlights the model’s stability and high-confidence prediction capability when multimodal cues are consistent.
2. **Case 2:** ***Padus avium*****—Robustness through Inter-Modal Compensation**
  In this case, the visual modality misclassifies the second query image due to significant intra-class visual variation between the support set (flower close-ups) and the query set (fruit close-ups). In contrast, the textual modality, guided by abstract semantic priors from structured prompts, remains invariant to such visual changes and provides a high-confidence correct prediction. Although the text branch is assigned a relatively low weight ($\alpha_{\mathrm{txt}}=0.27$), its dominant output determines the final fused result. This illustrates that the fusion mechanism can leverage high-confidence predictions from one modality to correct low-confidence errors from the other, highlighting the corrective role of the textual modality.
3. **Case 3:** ***Viburnum opulus*****—Validation of Adaptive Weighting Effectiveness**
  This case validates the effectiveness of adaptive weighting when the modalities exhibit divergent performance. In the second query image, the textual modality misclassifies the sample as *Padus avium*, while the visual modality maintains a relatively high-confidence prediction for the correct class. During weighted fusion, the image branch, with a significantly higher weight ($\alpha_{\mathrm{img}}=0.73$), dominates the decision, and the final prediction remains correct (*Viburnum opulus*). This demonstrates the fusion mechanism’s ability to anticipate modality reliability and prioritize the more trustworthy signal.

**Figure 14:**
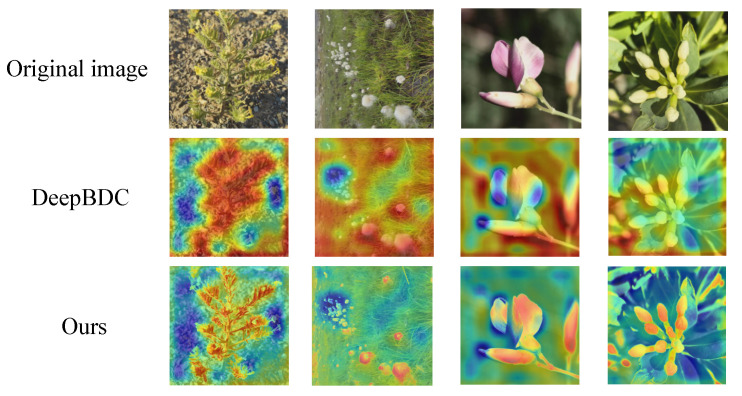
Visual comparison of feature activation maps. (**Top**) Original images. (**Middle**) Activation maps from the baseline DeepBDC, showing diffuse focus. (**Bottom**) Activation maps from our proposed model, demonstrating precise focus on discriminative local details and suppression of background interference.

To validate the effectiveness of the proposed multimodal feature fusion strategy within a deep metric learning framework, we compare the visualization results of DeepBDC [44] with those of the proposed BDCC model. As shown in Figure 14, the first row displays the original images, the second row presents the activation maps generated by DeepBDC [44], and the third row presents the activation maps produced by BDCC.

For DeepBDC [44], the activation maps are generated by registering a forward hook on the last convolutional layer of the network to capture the corresponding feature activation maps. These maps are then processed across the channel and spatial dimensions to obtain the final activation response maps. For BDCC, the activation maps are produced using the proposed fusion strategy: first, the activation maps from DeepBDC [44] and the gradient-based activation maps from the CLIP [15] model are each normalized; second, the adaptive weight strategy proposed in this work dynamically computes fusion coefficients based on support set performance, with an additional weight smoothing mechanism introduced to enhance stability; finally, the two normalized activation maps are combined via weighted fusion to generate an activation map that integrates both internal feature activation information and semantic gradient information. This results in a more accurate representation of the regions in the image that the model focuses on.

The results show that DeepBDC [44] tends to highlight the overall structure of the plant, whereas BDCC captures more discriminative local details—such as petal shapes and leaf textures—while effectively suppressing background interference.

#### 5.2.5. Analysis of Inference Speed and Practicality

To evaluate the deployment potential of the proposed method in real-world scenarios, the inference speed is quantitatively analyzed on a computer equipped with an NVIDIA GeForce RTX 4060 GPU.

The end-to-end inference time is measured under two core configurations: 5-way 1-shot and 5-way 5-shot. To ensure stability and reproducibility, five independent experimental runs are conducted, each involving 600 randomly generated tasks. The final metrics, including the mean and standard deviation, are obtained by averaging across these runs. The reported end-to-end time includes both the analysis of the support set for dynamic weight computation and the subsequent classification of all images in the query set. The results are summarized in Table 8.

The analysis indicates that our method is computationally efficient. In the 1-shot scenario, the model achieves a real-time throughput of approximately 29 FPS. While increasing the support samples to the 5-shot setting increases the task duration due to the larger volume of information to process, the model still maintains a near-real-time performance of approximately 9.5 FPS. This performance, especially on consumer-grade hardware, demonstrates that our proposed dynamic fusion strategy is both lightweight and effective.

More importantly, this provides the flexibility for practical applications to make a trade-off between speed and accuracy. Scenarios requiring a rapid response can opt for the 1-shot configuration, while those prioritizing higher accuracy can enhance performance by increasing the number of samples (e.g., to 5-shot). This adaptability significantly enhances the value of our method for field deployment in the domain of plant classification and recognition.

## 6. Conclusions

This paper introduces a new dataset and baseline method to address fine-grained plant image classification under data-scarce conditions in complex environments. Existing datasets are mostly collected under idealized settings, limiting the generalizability of current methods and leading to suboptimal performance in real-world applications.

To address this limitation, a new image dataset—FewMedical-XJAU—is presented, focusing on rare medicinal plants from Xinjiang. The images are captured in natural environments with diverse and complex backgrounds. The dataset exhibits significant intra-class variation and subtle inter-class differences, along with a wide range of shooting angles and lighting conditions, providing a realistic approximation of real-world deployment scenarios.

Although the proposed method demonstrates promising performance in fine-grained classification of medicinal plants with limited samples under complex real-world settings, it still relies on predefined structured textual prompts as input. Future work will explore automatic prompt generation strategies that incorporate highly domain-specific knowledge without the need for manually crafted inputs. This remains an open area of investigation.

## Figures and Tables

**Table 1:** Comparison of FewMedical-XJAU with existing plant datasets.

Dataset	Medicinal	Annotation Granularity *	Background Complexity **	Images	Species
Flavia [6]	No	1	1	1907	32
Swedish Leaf [9]	No	1	1	1125	15
LeafSnap [10]	No	1, 2	1, 2	30,966	185
ImageCLEF 2013 [7]	No	1, 2	1, 2	26,077	250
Pl@ntNet300K [8]	No	1, 2	2, 3	300,000	1081
Medicinal Leaf (MED117) [11]	Yes	1	1	77,700	117
Bangladeshi medicinal plant [12]	Yes	1	1	5000	10
DIMPSAR [13]	Yes	1	2	5900	40
Karst Landform Herbs [14]	Yes	2	2, 3	56,650	120
FewMedical-XJAU	Yes	3	2, 3	4992	540

* **Annotation Granularity:** The numbers in this column refer to: 1. *Flattened single-layer species labels:* A labeling standard based on plant species, employing a flattened, single-layer structure where each class corresponds to a unique species. 2. *Extended labels with organ-class metadata:* Labels are annotated through metadata files (e.g., XML or CSV), providing detailed information about the specific plant part in the image, such as leaf, flower, fruit, stem, branch, or the entire plant. 3. *Hierarchical taxonomic labeling system:* A hierarchical naming method is used, encoding the complete taxonomic path from phylum to species, along with corresponding identifiers, into the label system to reflect the systematic structure of plant classification. ** **Background Complexity:** The numbers in this column refer to: 1. *Plain white background:* Images are captured against a non-interfering, plain white background, such as on white paper or in a laboratory environment. 2. *Simple background:* The plant is situated in a relatively simple environment with no significant distractors, or the image is a close-up of a specific plant organ. 3. *Complex background:* The image contains significant distractors, such as other plants or rocks, or is captured with a wide-angle lens, encompassing a larger view of the plant and its natural habitat.

**Table 2:** Summary of representative studies in plant recognition.

Paper	Model	Dataset	Accuracy
Lee et al. (2017) [33]	AlexNet + MLP	MalayaKew(D2) ^dg^	99.50%
Lee et al. (2017) [33]	AlexNet + MLP	MalayaKew(D1) ^dg^	98.10%
Wei Tan et al. (2018) [34]	D-leaf	Flavia [6]	94.63%
Wei Tan et al. (2018) [34]	D-leaf	Swedish Leaf [9]	98.09%
Dudi and Rajesh (2022) [35]	SS-WOA-CNN ^a^	Swedish Leaf [9]	97.54%
Quach et al. (2023) [36]	SVM	Flavia [6]	99.69%
Tang et al. (2025) [14]	MPR-net	Karst Landform Herbs [14]	85.33%
Hoang et al. (2023) [37]	SENet + AHL ^b^	VietForest	81.14% (single) ^c^
Hoang et al. (2023) [37]	SENet + AHL ^b^	VietForest	89.35% (five) ^c^
Fang et al. (2024) [38]	CSDNet	SoyGene ^g^	86.86%
Fang et al. (2024) [38]	CSDNet	SoyLocal ^g^	60.50%
Fang et al. (2024) [38]	CSDNet	Cotton80 ^g^	67.92%
Arkel Rios et al. (2024) [39]	ViT + ILA ^e^	Cotton ^g^	55.42%
Arkel Rios et al. (2024) [39]	ViT + ILA ^e^	SoyGene ^g^	58.14%
Arkel Rios et al. (2024) [39]	ViT + ILA ^e^	SoyLocal ^g^	50.83%
Zou et al. (2023) [40]	S^2^CL-Leaf Net	Flavia [6]	75.30% (5-shot) ^f^
Zou et al. (2023) [40]	S^2^CL-Leaf Net	Swedish Leaf [9]	79.80% (5-shot) ^f^
Zou et al. (2023) [40]	S^2^CL-LeafNet	LeafSnap [10]	82.40% (5-shot) ^f^
Zulfiqar et al. (2025) [17]	FRN + Bi-FRN	Pl@ ntNet-300K [8]	77.42% (1-shot) ^f^
Zulfiqar et al. (2025) [17]	FRN + Bi-FRN	Pl@ ntNet-300K [8]	88.71% (5-shot) ^f^

^a^ SS-WOA-CNN: Salp Swarm Whale Optimization Algorithm-based Convolutional Neural Network. ^b^ AHL: Attentive Hierarchical Learning. ^c^ (single), (five): refers to accuracy based on single-crop vs. five-crop testing protocol. ^d^ D1: a subset of MalayaKew that contains only full leaves. D2: a subset of MalayaKew that contains only image patches from inside the leaves. ^e^ ILA: Instance-Level Augmentation. ^f^ (n-shot): refers to few-shot learning scenarios, where the model is trained with only “n” examples per class. ^g^ This dataset is not publicly available at the moment.

**Table 3:** The model information and accuracy results of classification training on the conventional experimental dataset.

Model	Year	Model Type	Epochs	Top-1 Acc	Top-5 Acc
ResNet-50 [28]	2015 (CVPR)	CNN	300	35.20	55.30
MAE [48]	2021 (CVPR)	CNN+ViT	300	39.71	58.8
MPViT_tiny [49]	2022 (CVPR)	CNN+ViT	300	43.77	52.18
SwiftFormer_XS [50]	2023 (ICCV)	CNN+ViT	300	5.99	13.08
SBCFormer_B [51]	2024 (WACV)	CNN+ViT	300	57.79	75.03
RepViT-M0.9 [52]	2024 (CVPR)	CNN+ViT	300	22.59	37.15
UniRepLKNet-A [53]	2024 (CVPR)	CNN+ViT	300	**74.61 **	**82.94 **
MPR-net [14]	2025 (PR)	CNN+ViT	300	68.75	81.25

The best and second best results are bold and underlined, respectively.

**Table 4:** Data splits for 5-way 1-shot and 5-way 5-shot tasks.

	5-Way 1-Shot	5-Way 5-Shot
Train	289 classes (2892 images)	181 classes (2392 images)
Validation	97 classes (971 images)	60 classes (786 images)
Test	97 classes (950 images)	62 classes (801 images)

**Table 5:** Performance comparison with state-of-the-art few-shot classification methods on the FewMedical-XJAU dataset.

Method	5-Way 1-Shot (%)	5-Way 5-Shot (%)
ProtoNet [23] (NeurIPS 2017)	57.88	69.50
DeepBDC [44] (CVPR 2022)	74.50	85.94
EM-Dirichlet [55] (CVPR 2024)	39.92	64.33
SRN [56] (PR 2024)	63.10	79.92
HMDRN [57] (arXiv 2025)	61.92	82.33
**BDCC (Ours)**	**83.02**	**89.15**

The best-performing results in the table are highlighted in bold.

**Table 6:** Accuracy comparison of ablation experiments. It is worth noting that BDCC employs the Simple Fixed-weight Fusion Strategy as its fusion method.

CLIP [15] (Zero-Shot Methods)		
Method	Top-1 Accuracy (%)	
CLIP [15] (“a photo of {class}”)	64.93	
CLIP [15] (Class-Aware Structured Text Prompt Construction)	71.35	
DeepBDC [44] and BDCC (Few-Shot Methods)		
Method	1-shot (%)	5-shot (%)
DeepBDC [44]	74.50	85.94
BDCC (“a photo of {class}”)	79.54	88.13
BDCC (Class-Aware Structured Text Prompt Construction)	**82.32 **	**88.94**

The best-performing results in the table are highlighted in bold.

**Table 7:** Ablation study of different fusion strategies. SFFS (Simple Fixed-weight Fusion Strategy), AWDC (Adaptive Weight Dynamic Fusion strategy based on Confidence), AWSS (Adaptive Weight Dynamic Fusion strategy based on Support Set Performance), Ours (Adaptive Weight based on Support Set Performance with Weight Smoothing). The 1-shot and 5-shot results are reported in %.

Fusion	Model Configuration	1-Shot	5-Shot
SFFS	BDCC (“a photo of class”)	79.54	88.13
	BDCC (Class-Aware Structured Text Prompt Construction)	82.32	88.94
AWDC [46]	BDCC (“a photo of class”)	78.73	82.89
	BDCC (Class-Aware Structured Text Prompt Construction)	81.99	83.32
AWSS [47]	BDCC (“a photo of class”)	80.13	88.13
	BDCC (Class-Aware Structured Text Prompt Construction)	81.99	88.53
Ours	BDCC (“a photo of class”)	80.24	88.53
	BDCC (Class-Aware Structured Text Prompt Construction)	**83.02**	**89.15**

The best-performing results in the table are highlighted in bold.

**Table 8:** Comparison of inference speed and performance under different few-shot configurations.

Metric	5-Way 1-Shot	5-Way 5-Shot
Average task time (ms)	342.89 ± 90.61	1048.34 ± 100.71
Average query image time (ms)	34.29	104.83
Throughput (FPS)	∼29	∼9.5

## Data Availability

The data supporting the findings of this study are available from the corresponding author upon reasonable request.
